# Arthroscopic osteochondroplasty in patients with mild slipped capital femoral epiphysis after in situ fixation

**DOI:** 10.1007/s11832-015-0707-3

**Published:** 2015-11-20

**Authors:** Philippe M. Tscholl, Patrick O. Zingg, Claudio Dora, Eric Frey, Stefan Dierauer, Leonhard E. Ramseier

**Affiliations:** Department of Orthopaedics, Balgrist University Hospital, University of Zurich, Forchstrasse 340, 8008 Zurich, Switzerland; Orthopaedic Division, Department of Surgery, University Children’s Hospital, University Hospital of Zurich, Zurich, Switzerland

**Keywords:** Slipped capital femoral epiphysis, Femoro-acetabular impingement, FAI, Osteochondroplasty, Magnetic resonance tomography, Osteoarthritis

## Abstract

**Purpose:**

Mild slipped capital femoral epiphyses (SCFE) nevertheless show significant femoral head–neck deformities which may put cartilage and acetabular labrum at risk. Whether this deformity can be restored to normal has not yet been described in the literature.

**Methods:**

In a prospective follow-up study, 14 patients with mild SCFE underwent in situ fixation with a single 6.5-mm cancellous, partially threaded screw. In 14 patients arthroscopic osteochondroplasty was performed, and in 13 patients pre- and postoperative measurements of the α-angle were made using antero-superior radial magnetic resonance imaging.

**Results:**

After arthroscopic osteochondroplasty, the mean α-angle decreased from 57° (range 50°–74°) to 37° (range 32°–47°; *p* < 0.001). Six patients showed beginning degenerative intra-articular changes (four antero-superior cartilage and three antero-superior labrum lesions) at the time of hip arthroscopy. No intra-operative complications occurred. In one patient, arthroscopic debridement was necessary due to arthrofibrosis and persistent pain.

**Conclusion:**

Arthroscopic osteochondroplasty can successfully correct the antero-superior α-angle in patients with mild SCFE to normal values. Clinical randomized controlled studies with long-term follow-up are required to find evidence of improved functional and radiographic mid- and long-term outcome compared to in situ fixation alone.

## Introduction

Slipped capital femoral epiphysis (SCFE) mainly occurs in young teenage boys, and is associated with overweight and endocrine disorders [1]. Its incidence is increasing, especially in younger children, and might even be under-reported due to its clinically silent course [1, 2]. SCFE is defined by a dorso-medial slippage of the proximal femoral epiphysis, leading to a change in the femoral head–neck axis, a femoro-acetabular pressure area and a consecutive femoral head–neck deformity antero-laterally. Goodman et al. [3] found a strong correlation between post-slip morphology (pistol-grip and femoral head tilt deformity) and osteoarthritis. He described “anterior flattening of the acetabulum, cystic degeneration in the anterior metaphyseal-epiphyseal region, and progression to global osteoarthritis”. This description corresponds to antero-superior femoro-acetabular impingement (FAI).

As described by Rab [4], the altered biomechanical femoro-acetabular contact area can lead to impaction-type or inclusion-type impingement. The latter is due to a prominence (bump) on the head–neck junction in mild SCFE [5] or to the remodelling process in higher grade SCFE resulting in a cam deformity. Impaction-type impingement is due to an abutment of the proximal metaphysis on the acetabular rim, which cannot enter the acetabulum as occurs in higher grade SCFE. Due to the hinge and lever effect on the acetabulum rim, increasing stress on the posterior acetabular cartilage is applied as well as direct antero-superior labral injuries [4]. Early degenerative changes with cartilaginous and labral injuries have been reported not only in moderate and severe but also in mild SCFE [3, 5–12].

Once mild SCFE is diagnosed, first-line therapy aims at decreasing further slippage without compromising epiphyseal vascularisation and chondrolysis [13] by in situ fixation with one or two cannulated, fully or long-threaded screws [14]. In moderate or severe SCFE open reduction and fixation may be indicated [15].

After in situ fixation of mild SCFE, cam deformity remains. We therefore conducted a prospective study of patients after in situ fixation suffering mild SCFE, and performed arthroscopic femoral neck osteochondroplasty. Our hypothesis was that by arthroscopic intervention, head–neck offset morphology could be restored to normal by comparing their pre- and postoperative α-angles on magnetic resonance imaging (MRI).

## Methods

Between January 2011 and August 2013, 23 patients presented with SCFE to the local university orthopaedic division. Following the Southwick classification of SCFE [16], eight patients with moderate to severe SCFE (>30°) were allocated to open reduction and fixation, and 15 patients with mild SCFE were allocated to percutaneous in situ fixation using a 6.5-mm cannulated screw [17]. On the contralateral side, in situ fixation by an identical method was performed prophylactically. Primary surgery was performed by two experienced paediatric orthopaedic surgeons. After in situ fixation the patients were referred for arthroscopic osteochondroplasty to the department of hip surgery. In 14 patients informed consent was given by their legal guardian before taking part in this study; one patient refused to take part in this study.

Ethical approval was obtained by the local ethical committee and the study was performed in accordance with the ethical standards as laid down in the 1964 Declaration of Helsinki and its later amendments.

### Hip arthroscopy

Hip arthroscopy was performed by two experienced orthopaedic hip surgeons. Standard antero-lateral and anterior portals were used for central assessment of labral and cartilaginous injuries and categorized with Beck’s classification [18]. An accessory distal mid-anterior portal was used to perform osteochondroplasty.

### Radiological measurements

**Fig. 1:**
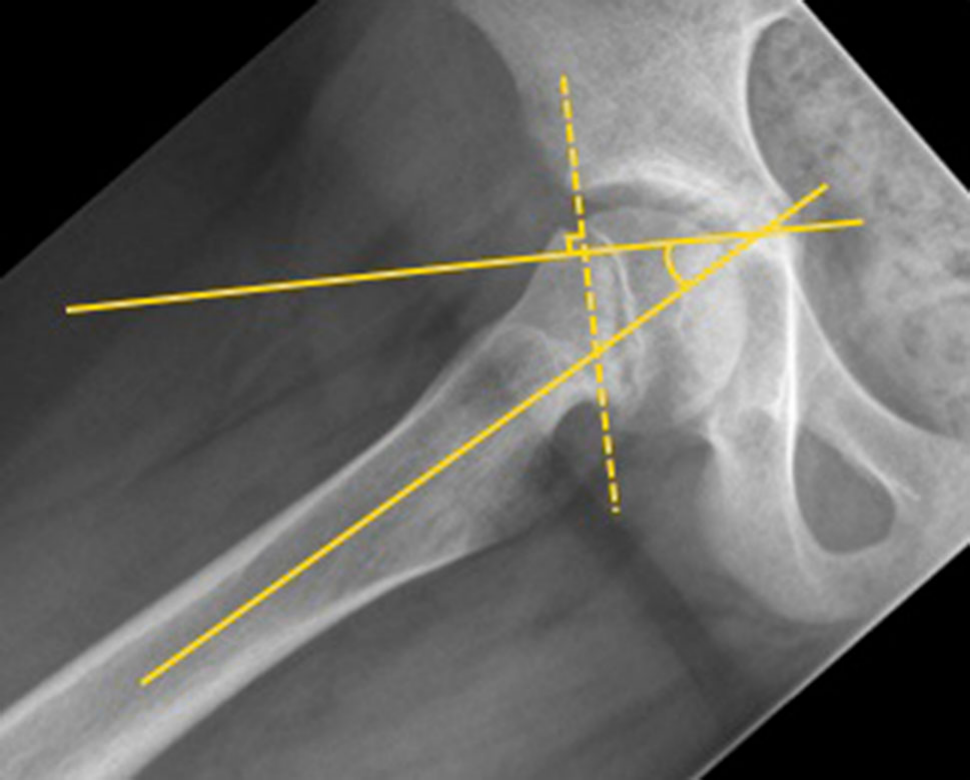
Measurement for head–shaft angle on conventional radiograph (patient no. 5) [16]

**Fig. 2:**
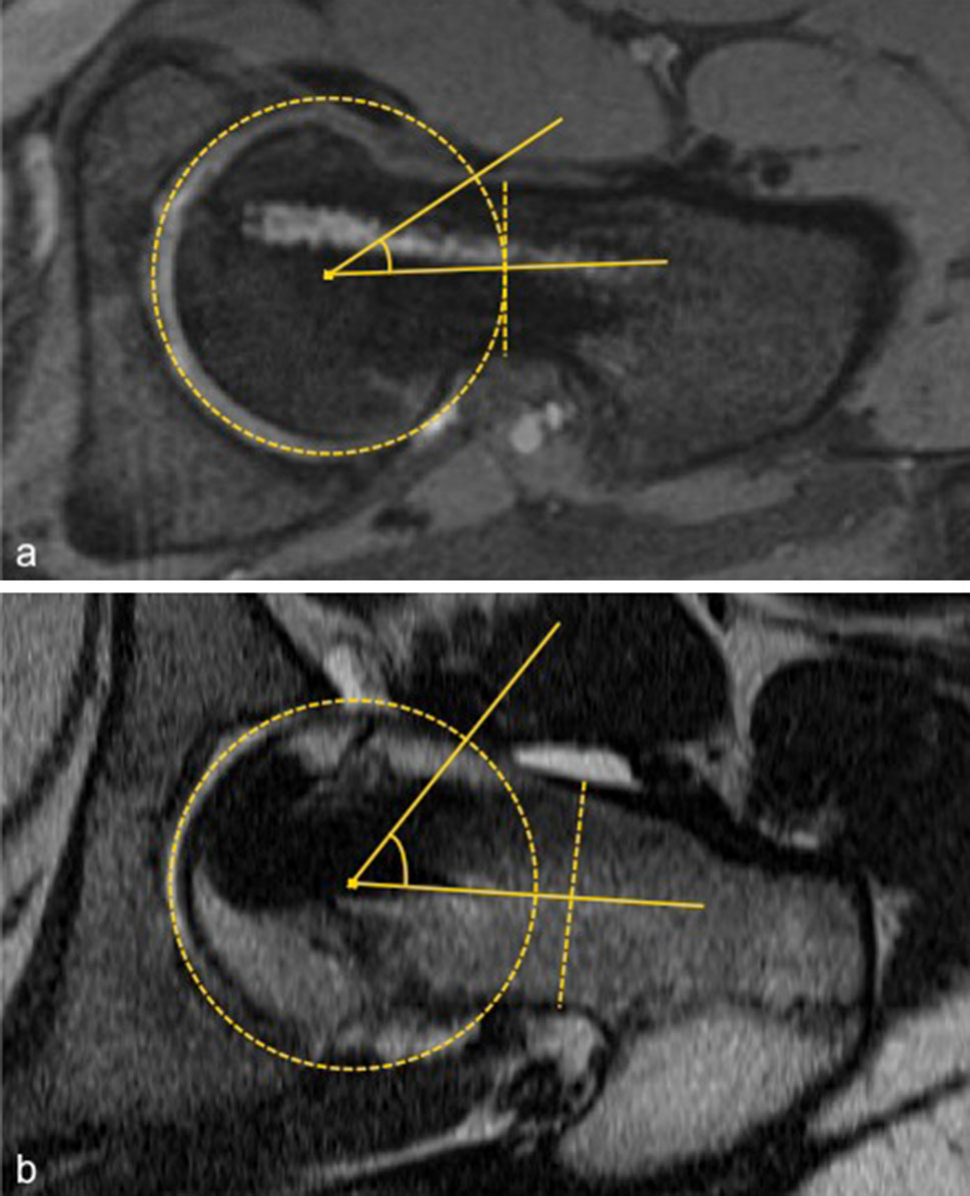
Alpha-angle on radial reformatted MR images (patient no. 1) **a** prior and **b** after hip arthroscopy [8, 19]

The head–shaft angle was measured as described by Southwick [16] on Lauenstein radiographs (Fig. 1). The α-angle was measured according to Nötzli et al. [8] on pre- and post-arthroscopic MRI (Fig. 2a). Radial reformatted MR images were used, since axial slices might underestimate the deformity [19, 20]. The largest α-angle between the anterior and superior position on the femoral neck was recorded and its position measured in degrees (the superior position being 0° and the anterior position 90°). The analysis of the α-angle was performed antero-superiorly, since this is the predominant localisation with the largest extent for femoral neck deformity in patients after SCFE [9, 19].

### Statistics

Inter-rater reliability was performed with intra-class correlation for single measures. Comparisons of means of non-parametric continuous data were performed using the Mann–Whitney *U* test. Significance was accepted at *p* < 0.05.

## Results

Fourteen patients (six female and eight male, 12.6 ± 1.1 years) were referred for arthroscopic osteochondroplasty: 13 with mild SCFE (mean head–shaft angle 16° ± 6°) and one patient with moderate SCFE, which was initially classified and referred as mild (Table 1). Arthroscopic osteochondroplasty was performed 3.7 weeks (range 0.7–14.1) after bilateral in situ fixation.

**Table 1:** Patient characteristics and radiographic measurements pre- and postoperatively

No.	Gender	BMI (kg/m^2^)	Hip	Head–shaft angle^a^	Age at hip arthroscopy (years)	Time interval ISF/HAS (weeks)	α-angle		
							Pre-op	Post-op	Correction
1	Female	29.3	Left	21°	11.7	6.1	69°	35°	34°
2	Female	17.5	Left	19°	10.8	1.1	52°	32°	20°
3	Female	23.3	Left	11°	13.1	1.3	54°	38°	16°
4	Female	22.4	Left	24°	11.6	1.1	74°	47°	27°
5	Female	28.4	Right	27°	11.7	0.7	51°	34°	18°
6	Female	22.3	Right	14°	12.6	1.4	60°	38°	22°
7	Male	23.1	Right	9°	12.6	1.0	55°	47°	8°
8	Male	25.1	Right	7°	13.9	2.3	51°	35°	16°
9	Male	20.8	Left	10°	11.3	8.8	53°	32°	21°
10	Male	23.9	Left	22°	15.1	14.1	58°	34°	23°
11	Male	23.1	Left	13°	12.4	1.0	59°	41°	18°
12	Male	23.4	Left	12°	12.9	4.6	50°	34°	16°
13	Male	22.3	Left	33°	14.6	6.1	56°	32°	24°
14	Male	32.3	Left	18°	12.9	1.4	−	42°	−

^a^According to Southwick [16]

*ISF* in-situ fixation, *HAS* hip arthroscopy

**Fig. 3:**
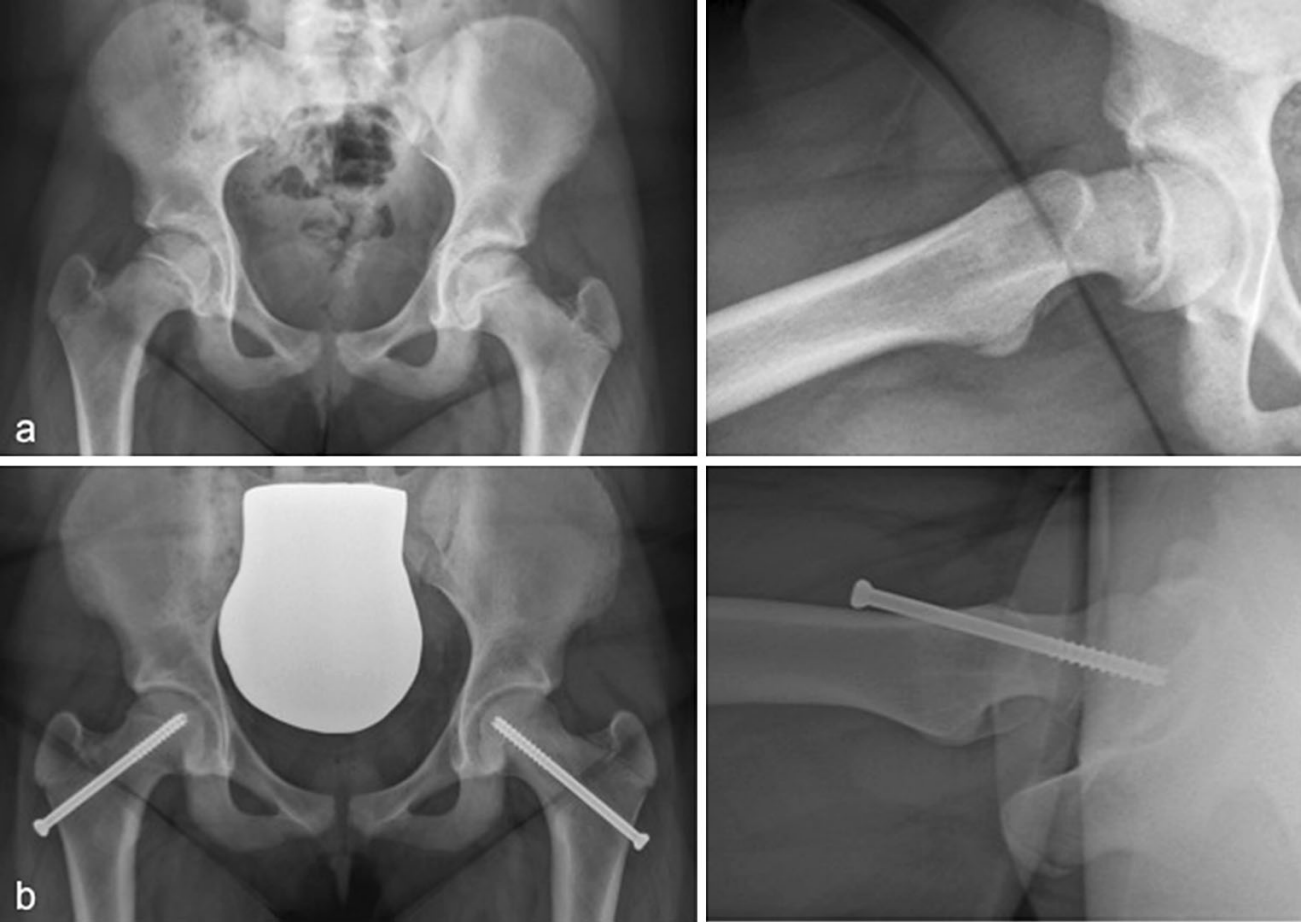
Antero-posterior and frog-leg radiograph prior (**a**) and after (**b**) in situ fixation and arthroscopic osteochondroplasty of the femoral neck (patient no. 13)

Measurements of α-angle were 25° ± 11° (range 5°–36°) on the clock model. Prior to arthroscopy, a mean α-angle of 57° ± 7° was found. A mean correction of 20° (range 8°–34°) at the antero-superior level could be achieved (Fig. 2), with all patients having a postoperative α-angle of less than 50° (Table 1). During arthroscopy, two grade I and two grade III cartilaginous injuries were found at the antero-superior level according to Beck’s classification. Antero-superior labral fraying was observed in three patients. None of these lesions required specific treatment except for slight debridement. No intra-operative complications occurred (Fig. 3). The patient with moderate SCFE requested arthroscopic debridement of arthrofibrosis 6 months after primary osteochondroplasty. No other complications occurred postoperatively, with a mean follow-up of 1.4 ± 0.7 years (0.9–3.4 years).

Inter-rater reliability of the Southwick angle (*r* = 0.97, *p* < 0.001), pre-operative α-angle (*r* = 0.96, *p* < 0.001) and postoperative α-angle (*r* = 0.86, *p* < 0.001) was excellent.

## Discussion

Our results show that the pathological α-angle measured at the antero-superior aspect of the femoral neck in patients who suffer SCFE can reliably be corrected by hip arthroscopy by an average of 20°. All postoperative α-angles were below 50°, which is considered normal [8, 9, 20]. Similar reductions of α-angle in patients with FAI but without a history of SCFE were described previously on cross-table, frog-leg, and antero-posterior radiographs after arthroscopic treatment [21].

In our cohort, four out of 14 patients showed beginning degeneration of the antero-superior acetabular cartilage and three patients of the antero-superior labrum in arthroscopy, which is in accordance with the literature [7, 22–24]. These still-asymptomatic lesions in our patients might become clinically evident years later and require secondary surgical treatment [25, 26].

Most studies agree that each grade of SCFE is equally associated with progression to hip osteoarthritis [23, 26], therefore early preventive intervention is desirable. In a retrospective study by Castaneda et al. [27] all patients, regardless of the severity of the initial epiphyseal slip—measured by the head–shaft angle—showed radiographic signs of osteoarthritis after a 22.3-year follow-up period; two out of three patients experienced pain when sitting or riding a bicycle and almost 90 % with the positive impingement test. On the other hand, some patients show satisfactory long-term outcome in their hip function even after severe head–neck deformity after SCFE without any surgical treatment [28–30]. This might to some extent be due to partial spontaneous remodelling of the proximal femoral epiphysis [30–32]. Most interestingly, in situations of pre-slip or prophylactic in situ fixation, Dodds et al. found no clinical hip impingement, leading to the conclusion that only slip leads to femoro-acetabular impingement [26]. Whether early arthroscopic osteochondroplasty is superior to a more expectant approach to correcting femoral head–neck deformity when epiphyseal growth is finished is unknown.

**Fig. 4:**
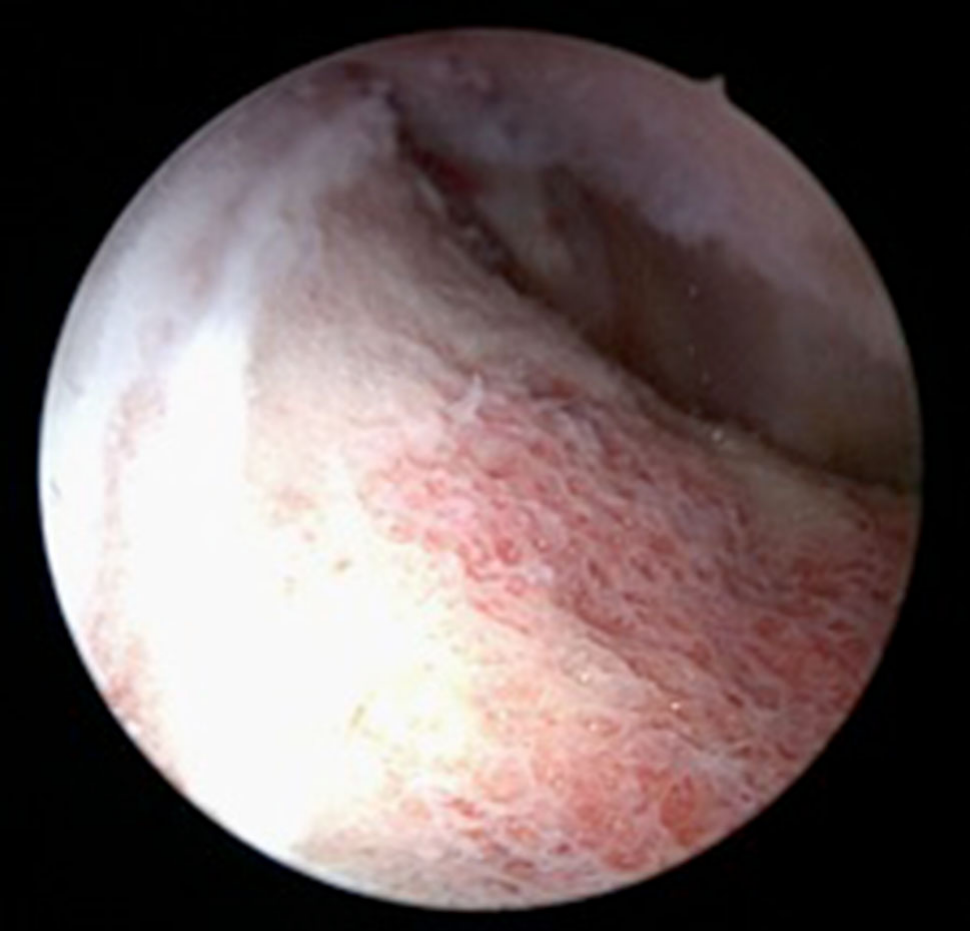
Arthroscopic antero-superior view of the femoral head–neck junction after trimming. Notice the *white line*, corresponding to the epiphyseal growth plate (patient no. 2)

Since the most pronounced deformity is found at the antero-superior and superior level [9], corrective osteochondroplasty is easily accessible by arthroscopy using standard portals (Fig. 4), [9, 33]. Except for one arthroscopic re-intervention due to arthrofibrosis 6 months after osteochondroplasty, no complications occurred. It can be assumed that an additional arthroscopic remodelling of the femoral neck after in situ fixation in patients with mild SCFE is a safe procedure, at least in the short term, that can restore the α-angle. Hence it might have the potential, as in patients with hip impingement, to reduce the early onset of symptoms and improve hip function and functional activity [9, 34, 35]. Whether one- or two-stage surgery should be favoured is unknown [24].

Restoring the α-angle in patients with moderate SCFE might also be possible, as shown for patient 13 (Table 1). The number of patients, however, is too small to state whether arthroscopic osteochondroplasty in patients with a head–shaft angle of more than 30° is contraindicated. In moderate and severe SCFE, an open surgical approach such as the (modified) Dunn osteotomy is indicated, with only low incidence of avascular necrosis of the femoral head [15, 36–38], or other inter- or subtrochanteric osteotomies [39].

It has been reported in the literature that patients with SCFE seem to have a higher incidence of acetabular and femoral retroversion, coxa profunda and reduced femoral head–neck offset [40, 41]. Hence, femoral head–neck deformity due to SCFE may only be a co-factor in such patients, resulting in symptomatic FAI [5]. Osteochondroplasty might therefore not be sufficient to prevent further acetabular cartilage and labral injury in all patients and additional interventions might be indicated [42]. Further studies need to be performed.

In conclusion, cam impingement deformity is found in patients with mild SCFE and, as in our cohort, is associated with antero-superior cartilage and labral injury. These lesions might be prevented if early osteochondroplasty is performed. Arthroscopic trimming is a safe and efficient surgical option for treating mild SCFE-related FAI, and can restore the α-angle to normal. Whether combined arthroscopic osteochondroplasty with in situ fixation improves long-term hip function and prevents progression of osteoarthritis after SCFE is unknown, and needs to be proven in prospective studies with long-term outcomes. However, in our understanding of FAI and its implications for osteoarthritis, early treatment, especially in patients with SCFE, is crucial to reduce the incidence of labral and acetabular degenerative lesions, and can be performed as two-stage or concomitant surgery after in situ fixation.
